# Boston Letter

**Published:** 1882-05

**Authors:** 


### Domestic Correspondence.

ARTICLE XIII.

MESSRS. EDITORS: In former letters, I believe, you have been made acquainted with the fact that the Massachusetts Medical Society, for purposes of local meetings and work, was long ago broken up into branch societies, according to counties. The main society meets but once yearly. The Boston branch is called the Suffolk District Society, and formerly met once per month. An effort was made last year to sustain fortnightly meetings. This did not work. The present secretary of the District Society, Dr. H. C. Haven, then proposed a division of the society into sections. Members might have the privilege of joining either or all of them. The sections thus far established are the Surgical and the Section for Clinical Medicine, Therapeutics and Pathology. Dr. Haven has pushed the matter with such vigor that the meetings of these two sections have become more interesting and active than the former meetings of the whole ever were.

The sections were to have met once in six weeks, but such is the interest already developed that the accumulated medical papers are too many for the number of meetings appointed. The order, therefore, has been changed, and the sections will meet oftener. The success of this arrangement simply shows that while men will not regularly attend meetings at which any medical topic is in order, they will closely follow up meetings at which they are sure to hear something directly in the line of their work. Both the surgical and clinical sections are well attended and extremely attractive. The general meetings of the District Society occur at regular intervals, but not so frequently as formerly. These occasions are followed by an abundant collation, which, it must be confessed, is enjoyed by many who fail to appear at the sections. Does this happen to suggest to you that these men choose the meeting at which they also are sure to encounter something directly in the line of their thought?

I think Dr. Haven may not realize how earnestly his efforts in bringing about section work and increased activity are appreciated by the members of the sections. So far as my knowledge goes, this splitting of a local society into special divisions has never before been attempted in this State. The arrangement is far more enjoyable than the old plan for reasons which are so obvious as to require no mention.

During the past winter we have had such share of the small-pox epidemic as could find admission in the existence of the unlimited power of our Board of Health. During the year 1881 eight deaths occurred in Boston from this disease. Since the 1st of January there have been a few additional deaths. The desire for vaccination has been wide-spread. The number of persons vaccinated by the city physician has been large, and private physicians have all done the operation many times. During a few weeks of the early winter the people at large were considerably anxious in regard to what was believed to be imminent danger. This feeling, however, has subsided. In one of the suburbs we have an excellent small-pox hospital, capable of accommodating seventy-five patients. Far from being forbidding in its appearance, it resembles, in the approving language of a former patient, "a summer hotel." It has an inviting parlor, attractively furnished, and containing an organ and piano, convenient bath-rooms, and is exceedingly comfortable, especially in the rooms designed for private patients. The kitchen department is well managed. During the winter the city physician has made daily visits. Free patients are as kindly treated as the private. But little medicine is given. Large quantities of milk are drank. Dieting and rest are the main treatment. But five or six patients have been under treatment at one time. The attractions and comforts of the place render it not only inviting, even to those who are obliged to leave luxurious homes, but better treatment is secured here than would be possible elsewhere. In reading a very old book on inoculation, written during the last century, I came upon an account of a curious and amusing phillipic pronounced in 1722 by a London clergyman against the practice of inoculation. Partisan feeling ran very high at the time, and the redoubtable parson thundered "Against the sinful and dangerous practice of inoculation." He called inoculators "diabolical sorcerers, hellish *Venesici*, enemies of mankind." With the odd superstition so common in that day he denounced inoculation as "a very ancient art, first put in practice upon Job by the devil." "Thus we are to understand," says a sarcastic cotemporary, "that the devil was the first inoculator, and poor Job his first patient." The doughty parson further asserted that inoculation was "an anti-providential project which insulted religion and banished Providence out of the world." A magazine of the day, referring to the conceit of the witty minister, published the following couplet:

"We're told by one of the black robe,
The devil inoculated *Job.*
Suppose 'tis true, what he does tell,
Pray, neighbors, did not *Job do well?*"

The Chinese, according to this interesting book, inoculated in very ancient times, and perhaps still do, by taking three or four scabs from a small-pox patient, and placing them between a bit of musk, wrapped the whole in cotton wool. This they inserted within the nostril of the subject; into the left nostril, if a male; into the right nostril, if a female. A method which proved largely unsuccessful.

The Greeks superstiously inoculated the forehead, each arm and the breast, in order thus to secure the form of the cross. Many other curious facts are related. Inoculation undoubtedly proved a blessing to those upon whom it was practiced, but with as little doubt it is responsible for the endemic existence of the disease in Asia at the present day, especially in China, where the custom is still in force.

It is a noteworthy fact that while in Great Britain inoculation had barely secured a foothold, in Boston and other neighboring New England towns, Dr. Zabdiel Boylston, incited by the publication of the method by Cotton Mather, inoculated 280 persons between June, 1721, and the following January.

The report of the trustees of the Massachusetts General Hospital for 1881 includes the valuable statement that the public has contributed the needed sum of $100,000 for the erection and support of a Convalescent Home, a dependency of the hospital. The building, well arranged and picturesque, will be ready for occupancy this spring. It will add greatly to the curative resources of the hospital.

The trustees touch upon another matter which dire necessity has made very urgent, viz.: The need of a hospital for incurables. While every provision has been made for acute diseases and accidents, so soon as a patient proves incurable, he has no other refuge than the discomforts of his own home. Proper surroundings and good care, on the other hand, would make the remaining years of his life comparatively free from suffering. Probably no physician in Boston, who does charitable work, but has found himself unable to provide for incurable patients. This is a sad need, and the trustees of the Massachusetts Hospital have done wisely in calling for donations and legacies to be applied in establishing a department for this class of sufferers. The practice of exacting a nominal fee from all applicants (excepting the very poor) to the out-patient department, and which, as I wrote you twelve months ago, was under trial, was soon abandoned. It failed to accomplish good results, and really produced evil. In April last a person of experience was appointed to examine all applicants, in order to discover whether they were entitled to gratuitous service. Of 1,250 cases visited at given addresses, 545 were found undeserving of charitable aid. To a large number of these, however, the trustees think it would be unjust to apply the term "impostor." They feel that the aim of such investigations should be rather to prevent than detect improper applications, and very sensibly add that although the result may in some degree diminish the number of those annually treated, "it is well to remember that to judge of the success of an institution by the number of its patients, is to apply the easiest but not the most adequate test."

Another effort has just been made to open the doors of the Harvard Medical School to women. The basis of the endeavor was an oger of $60,000, the gift being conditioned upon the admission of women to the Medical Department of the University. If sufficient space remained, I would give you full details of this new attack. In a certain sense it was sprung upon the medical faculty. They were not prepared for it. But they did a heap of work in a very short space of time, and the result is that today the University Committee upon the Medical Education of Women presented its report to the overseers of the college. It was discussed for two hours, and then, by a dangerously close vote (13 to 12), it was decided that "in the opinion of this board it is not advisable for the University to give any assurance or hold ont any encouragement that it will undertake the medical education of women by Harvard College in its medical school." So mote it be.

Boston, April, 1882. * *

